# A new form of Cd_3_TeO_6_ revealing dimorphism

**DOI:** 10.1107/S2056989018014214

**Published:** 2018-10-12

**Authors:** Matthias Weil, Théo Veyer

**Affiliations:** a Institute for Chemical Technologies and Analytics, Division of Structural Chemistry, TU Wien, Getreidemarkt 9/164-SC, A-1060 Vienna, Austria; b IUT Bordeaux 1, 15 Rue Naudet, 33175 Gradignan, France

**Keywords:** crystal structure, dimorphism, Mg_3_TeO_6_ structure type, solid solution

## Abstract

Phase-formation studies in the system CdO–TeO_3_ using a CsCl/NaCl melt at comparatively low temperatures revealed that tricadmium orthotellurate(VI), Cd_3_TeO_6_, is dimorphic. The new modification of Cd_3_TeO_6_ is denoted as the β-form and adopts the rhombohedral Mg_3_TeO_6_ structure type with one Cd and two O sites in general positions, and two Te sites with site symmetry  each. In comparison with the previously reported monoclinic cryolite-type α-form that was prepared at higher temperatures, β-Cd_3_TeO_6_ has a much lower density and most likely represents a metastable modification. Whereas the [TeO_6_] octa­hedra in both polymorphs are very similar and show only minor deviations from ideal values, the polyhedra around the Cd^II^ sites are different, with a distorted [CdO_6_] octa­hedron in both modifications but an additional [CdO_8_] polyhedron with a [4 + 4] coordination in the α-form.

## Abstract 2

A new modification of Cd_3_TeO_6_, denoted as the β-form, has been structurally determined, adopting the rhombohedral Mg_3_TeO_6_ structure type.

## Chemical context   

Various salts of *meta-*telluric acid, H_2_TeO_4_, have been reported as a result of high-pressure and high-temperature experiments (3000 atm; 973 K) aiming at various *M* ^II^TeO_4_ phases, where *M* = Mg, Ca, Sr, Ba, Cd or Pb (Sleight *et al.*, 1972 ▸). Meanwhile, the crystal structures of the Ca, Sr and Ba salts were determined (Hottentot & Loopstra, 1979 ▸; Weil *et al.*, 2016 ▸) whereas those of the other phases remain unknown to date. In a recent project on single-crystal growth of the Cd salt of *meta-*telluric acid, we used a CsCl/NaCl salt mixture (Źemcźuźny & Rambach, 1909 ▸) at temperatures < 800 K as a flux. Instead of the target phase CdTeO_4_, we obtained a new form of Cd_3_TeO_6_. The previously reported Cd_3_TeO_6_ polymorph crystallizes as a monoclinically distorted cryolite-type material in space-group type *P*2_1_/*n* (Burckhardt *et al.*, 1982 ▸) while the new form adopts the rhombohedral Mg_3_TeO_6_ structure type.

Prior to the current study, solid solutions Cd_3–*x*_Mn_*x*_TeO_6_ with *x* = 3, 2, 1.5 and 1 were prepared in polycrystalline form (Ivanov *et al.*, 2012 ▸), but not the cadmium end member, *i.e*. where *x* = 0. We report here the crystal structure of the new polymorph of Cd_3_TeO_6_, together with a comparative discussion of isostructural solid solutions Cd_3–*x*_Mn_*x*_TeO_6_. In the following, we refer to the previously reported monoclinic polymorph of Cd_3_TeO_6_ (Burckhardt *et al.*, 1982 ▸) as the α-form, and the new rhombohedral polymorph as the β-form of Cd_3_TeO_6_.

## Structural commentary   

**Figure 1:**
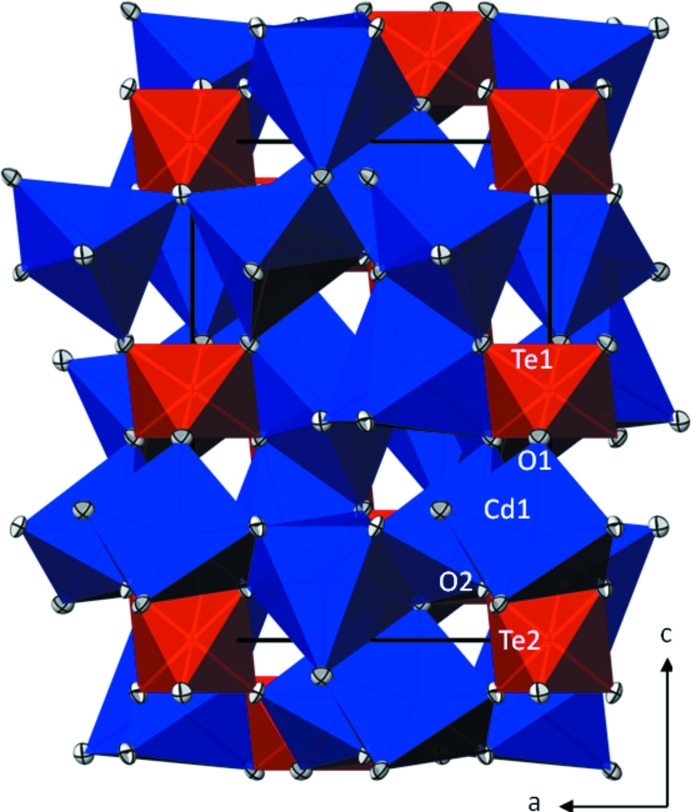
The crystal structure of β-Cd_3_TeO_6_ in polyhedral view in a projection along [0
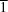
0]. [CdO_6_] octa­hedra are blue and [TeO_6_] octa­hedra are red. Displacement ellipsoids are drawn at the 90% probability level.

The crystal structure of β-Cd_3_TeO_6_ (Fig. 1 ▸) is made up from a distorted close packing of hexa­gonal oxygen layers extending parallel to (001). The Cd site (site symmetry 1) and the two unique Te sites (each with site symmetry 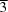) are situated in the octa­hedral inter­stices of this arrangement. The distorted [CdO_6_] octa­hedron has Cd—O distances ranging from 2.2348 (17)–2.4658 (19) Å (Table 1 ▸) and shares one edge with a [Te1O_6_] octa­hedron, another edge with a [Te2O_6_] octa­hedron, and four edges with neighbouring [CdO_6_] octa­hedra. Both [TeO_6_] octa­hedra show only minute deviations from the ideal octa­hedral symmetry. They are isolated from each other and are connected to six [CdO_6_] octa­hedra by sharing edges. The average Te—O bond length in β-Cd_3_TeO_6_ (1.931 Å) is in very good agreement with the mean Te—O bond length of 1.923 Å calculated for numerous (> 100) oxotellurates with octa­hedrally coordinated Te^VI^ (Christy *et al.*, 2016 ▸; Gagné & Hawthorne, 2018 ▸). Both unique O atoms are bonded to one Te and three Cd atoms in the form of a distorted tetra­hedron.

Like β-Cd_3_TeO_6_, Mn_3_TeO_6_ (Weil, 2006 ▸) as well as phases with *x* = 2, 1.5 and 1 of the Cd_3–*x*_Mn_*x*_TeO_6_ solid-solution series (Ivanov *et al.*, 2012 ▸) adopt the rhombohedral Mg_3_TeO_6_ structure type. A comparison of the bond lengths of the [*M*O_6_] (*M* = Cd, Mn) octa­hedra in the end members β-Cd_3_TeO_6_ and Mn_3_TeO_6_ and the solid solution Cd_1.5_Mn_1.5_TeO_6_ (mixed occupancy for the *M* site) shows inter­mediate values for the solid solution, consistent with the different ionic radii for six-coordinate Cd^II^ and Mn^II^ of 0.95 and 0.83 (high-spin) Å, respectively (Shannon, 1976 ▸). For a qu­anti­tative structural comparison of the end members β-Cd_3_TeO_6_ and Mn_3_TeO_6_ the program *compstru* (de la Flor *et al.*, 2016 ▸) available at the Bilbao Crystallographic Server (Aroyo *et al.*, 2006 ▸) was used. The degree of lattice distortion is 0.0204, the maximum distance between the atomic positions of paired atoms is 0.0680 Å for pair O2, the arithmetic mean of all distances is 0.0417 Å, and the measure of similarity is 0.011. All these values show a high similarity between the two crystal structures.

The structure of the monoclinic α-form of Cd_3_TeO_6_ (Burckhardt *et al.*, 1982 ▸) comprises of two cadmium sites (one on a general position and one on an inversion centre), one tellurium site on an inversion centre and three oxygen sites in general positions. While the [TeO_6_] octa­hedra in both Cd_3_TeO_6_ polymorphs have nearly the same bond length distribution [2 × 1.904 (4), 2 × 1.924 (5), 2 × 1.948 (4) Å in the α-form; for the β-form, see: Table 1 ▸], the set of coordin­ation polyhedra around the two Cd^II^ cations in the two structures is different. In β-Cd_3_TeO_6_, the cadmium site has a coordination number (CN) of six with an octa­hedral oxygen environment whereas in α-Cd_3_TeO_6_, only one site is octa­hedrally surrounded [range of Cd—O bond lengths: 2.211 (5)–2.350 (4) Å] and the other site exhibits a distorted [4 + 4] coordination [range of Cd—O bond lengths: 2.237 (5)–3.010 (5) Å].

As noted above, the end members β-Cd_3_TeO_6_ and Mn_3_TeO_6_ crystallize in the same structure type, suggesting a full miscibility over the complete range of *x* for the solid-solution series Cd_3–*x*_Mn_*x*_TeO_6_. However, the adopted structure type for the complete range of *x* appears to be dependent on the reaction temperature. Single crystals of α-Cd_3_TeO_6_ for structure analysis were grown from a 9 CdO: 11 TeO_2_ mixture that was heated in air at 1350 K for three h (Burckhardt *et al.*, 1982 ▸) while single crystals of β-Cd_3_TeO_6_ were obtained at much lower temperatures (793 K) using a flux method. This suggests that the high-temperature synthesis yields the thermodynamically stable modification. The rule of thumb that in the majority of cases the denser polymorph represents also the thermodynamically stable modification supports this assumption because α-Cd_3_TeO_6_ [*D_x_* = 7.490 (2) g cm^−3^; Burckhardt *et al.*, 1982 ▸] is much denser than β-Cd_3_TeO_6_ [*D_x_* = 6.941 g cm^−3^]. Under consideration of the similar reaction conditions for preparation of monoclinic α-Cd_3_TeO_6_ and the given solid solutions Cd_3–*x*_Mn_*x*_TeO_6_ (1270 K following a ceramic route; Ivanov *et al.*, 2012 ▸), it appears likely that the rhombohedral β-Cd_3_TeO_6_ end member can be prepared only at lower temperatures whereas certain amounts of manganese substituting cadmium in the Cd_3–*x*_Mn_*x*_TeO_6_ solid-solution series stabilize the Mg_3_TeO_6_ structure type at higher temperatures. Unfortunately, because of the scarcity of β-Cd_3_TeO_6_ material, a detailed investigation of the thermal behaviour of this phase, *e.g*. in terms of stability and a possible phase transition to α-Cd_3_TeO_6_, could not be undertaken.

## Database survey   

According to a search of the Inorganic Crystal Structure Database (ICSD; Belsky *et al.*, 2002 ▸), the Mg_3_TeO_6_ structure type is realized for eponymous Mg_3_TeO_6_ (Schulz & Bayer, 1971 ▸), Ca_3_UO_6_ (Holc & Golic, 1983 ▸), Mn_3_WO_6_ (Klüver & Müller-Buschbaum, 1994 ▸), Li_3_AlD_6_ (Brinks & Hauback, 2003 ▸; Løvvik *et al.*, 2004 ▸), Mn_3_TeO_6_ (Weil, 2006 ▸), selected solid solutions Cd_3–*x*_Mn_*x*_TeO_6_ (Ivanov *et al.*, 2012 ▸), Mn_3-*x*_Co_*x*_TeO_6_ (Singh *et al.*, 2014 ▸; Ivanov *et al.*, 2014 ▸), Mn_2.4_Cu_0.6_TeO_6_ (Wulff *et al.*, 1998 ▸), (Ca_0.2667_ Y_0.7333_)_3_(Y_0.2_Sn_0.3_)Sn_0.5_O6 (Kaminaga *et al.*, 2006 ▸), Mn_2_InSbO_6_ and Mn_2_ScSbO_6_ (Ivanov *et al.*, 2011 ▸), Sc_3_(Sc_0.295_ Al_0.705_)O_6_ (Müller *et al.*, 2004 ▸) and Ho_3_ScO_6_ (Badie, 1973 ▸).

## Synthesis and crystallization   

The rhombohedral β-form of Cd_3_TeO_3_ was obtained as one of the products from a flux synthesis using a CsCl/NaCl salt mixture (molar ratio 0.65/0.35). To 1.7 g of the salt mixture were added CdO (0.13 g) and TeO_3_ (0.18 g). TeO_3_ had previously been prepared by heating H_6_TeO_6_ at 573 K for 8 h. The reaction mixture was evacuated and sealed in a silica ampoule, heated from room temperature within 3 h to 793 K, kept at that temperature for 90 h and cooled within 10 h back to room temperature. The silica ampoule was subsequently broken and the solidified melt leached out with water for 2 h. The off-white product was filtered off, washed with water and was air-dried. The title compound was present in the form of a few nearly spherical colourless crystals. Other phases identified by single-crystal X-ray diffraction measurements of selected crystals were α-Cd_3_TeO_6_ (Burckhardt *et al.*, 1982 ▸), the mixed-valent Te^IV/VI^ compound Cd_2_Te_2_O_7_ (Weil, 2004 ▸) and a new form of incommensurately modulated CdTe_2_O_5_ (Weil & Stöger, 2018 ▸). Estimated on optical inspection with a microscope, all these phases represent minor by-products. Powder X-ray diffraction measurements of the bulk additionally revealed triple-perovskite-type CsCdCl_3_ (Siegel & Gebert, 1964 ▸) as the main phase and the Te^IV^ compound CdTeO_3_ (Krämer & Brandt, 1985 ▸) as a minority phase. Some additional reflections in the X-ray powder diffraction pattern of the bulk could not be assigned to the phases mentioned above or to any other known phase(s).

## Refinement   

Crystal data, data collection and structure refinement details are summarized in Table 2 ▸. Standardized coordinates (Gelato & Parthé, 1987 ▸) from the isotypic phase Mn_3_TeO_6_ (Weil, 2006 ▸) were taken as starting parameters for refinement. The highest and lowest remaining electron density peaks are located 1.56 and 1.53 Å from sites Te2 and O1, respectively.

## Supplementary Material

Crystal structure: contains datablock(s) I, global. DOI: 10.1107/S2056989018014214/vn2137sup1.cif

Structure factors: contains datablock(s) I. DOI: 10.1107/S2056989018014214/vn2137Isup2.hkl

CCDC reference: 1872058

Additional supporting information:  crystallographic information; 3D view; checkCIF report

## Figures and Tables

**Table 1:** Selected bond lengths (Å) in rhombohedral β-Cd_3_TeO_6_ and in isotypic (Cd_1.5_Mn_1.5_)TeO_6_ and Mn_3_TeO_6_

	β-Cd_3_TeO_6_^*a*^	Cd_1.5_Mn_1.5_TeO_6_^*b*^	Mn_3_TeO_6_^*c*^
*M*1—O1	2.2348 (17)	2.147	2.1055 (14)
*M*1—O2^i^	2.2455 (17)	2.150	2.1275 (13)
*M*1—O1^ii^	2.2907 (19)	2.240	2.2009 (13)
*M*1—O2^iii^	2.3051 (18)	2.260	2.2311 (12)
*M*1—O2	2.3370 (18)	2.273	2.2313 (13)
*M*1—O1^iv^	2.4658 (19)	2.412	2.3841 (13)
Te1—O1	1.9339 (17)	1.955	1.9247 (13)
Te2—O2	1.9290 (17)	1.959	1.9214 (12)

Notes: (*a*) This study; (*b*) Ivanov *et al.* (2012 ▸) on the basis of X-ray powder diffraction data at room temperature (no s.u. given in original publication); (*c*) Weil (2006 ▸) on the basis of single-crystal X-ray data at room temperature. [Symmetry codes: (i) *y* − 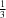, −*x* + *y* + 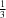, −*z* + 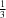; (ii) −*x* + 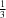, *y* + 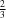, −*z* + 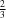; (iii) −*y*, *x* − *y*, *z*; (iv) −*y* + 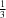, *x* − *y* + 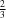, *z* − 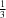.]

**Table 2:** Experimental details

Crystal data	
Chemical formula	Cd_3_TeO_6_
*M*_r_	560.80
Crystal system, space group	Trigonal, *R* 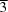 :*H*
Temperature (K)	296
*a*, *c* (Å)	9.1620 (2), 11.0736 (3)
*V* (Å^3^)	805.01 (4)
*Z*	6
Radiation type	Mo *K*α
μ (mm^−1^)	17.06
Crystal size (mm)	0.08 (radius)

Data collection	
Diffractometer	Bruker APEXII CCD
Absorption correction	Multi-scan (*SADABS*; Krause *et al.*, 2015 ▸)
*T*_min_, *T*_max_	0.527, 0.749
No. of measured, independent and observed [*I* > 2σ(*I*)] reflections	11351, 1623, 1526
*R*_int_	0.033
(sin θ/λ)_max_ (Å^−1^)	1.025

Refinement	
*R*[*F* ^2^ > 2σ(*F* ^2^)], *wR*(*F* ^2^), *S*	0.023, 0.046, 1.29
No. of reflections	1623
No. of parameters	33
Δρ_max_, Δρ_min_ (e Å^−3^)	2.57, −1.53

Computer programs: *APEX3* and *SAINT* (Bruker, 2015 ▸), *SHELXL2017/1* (Sheldrick, 2015 ▸), *ATOMS for Windows* (Dowty, 2006 ▸) and *publCIF* (Westrip, 2010 ▸).

## supplementary crystallographic information

### Crystal data

**Table:**

Cd_3_TeO_6_	*D*_x_ = 6.941 Mg m^−^^3^
*M**_r_* = 560.80	Mo *K*α radiation, λ = 0.71073 Å
Trigonal, *R*3:*H*	Cell parameters from 6637 reflections
*a* = 9.1620 (2) Å	θ = 5.0–46.6°
*c* = 11.0736 (3) Å	µ = 17.06 mm^−^^1^
*V* = 805.01 (4) Å^3^	*T* = 296 K
*Z* = 6	Spherical, colourless
*F*(000) = 1464	0.08 × 0.08 × 0.08 × 0.08 (radius) mm

### Data collection

**Table:**

Bruker APEXII CCD diffractometer	1526 reflections with *I* > 2σ(*I*)
ω– and φ–scans	*R*_int_ = 0.033
Absorption correction: multi-scan (*SADABS*; Krause *et al.*, 2015)	θ_max_ = 46.8°, θ_min_ = 3.2°
*T*_min_ = 0.527, *T*_max_ = 0.749	*h* = −18→18
11351 measured reflections	*k* = −18→16
1623 independent reflections	*l* = −22→22

### Refinement

**Table:**

Refinement on *F*^2^	0 restraints
Least-squares matrix: full	*w* = 1/[σ^2^(*F*_o_^2^) + (0.0021*P*)^2^ + 11.2674*P*] where *P* = (*F*_o_^2^ + 2*F*_c_^2^)/3
*R*[*F*^2^ > 2σ(*F*^2^)] = 0.023	(Δ/σ)_max_ < 0.001
*wR*(*F*^2^) = 0.046	Δρ_max_ = 2.57 e Å^−^^3^
*S* = 1.29	Δρ_min_ = −1.53 e Å^−^^3^
1623 reflections	Extinction correction: SHELXL-2017/1 (Sheldrick 2015), Fc^*^=kFc[1+0.001xFc^2^λ^3^/sin(2θ)]^-1/4^
33 parameters	Extinction coefficient: 0.00434 (9)

### Special details

**Table:**

Geometry. All esds (except the esd in the dihedral angle between two l.s. planes) are estimated using the full covariance matrix. The cell esds are taken into account individually in the estimation of esds in distances, angles and torsion angles; correlations between esds in cell parameters are only used when they are defined by crystal symmetry. An approximate (isotropic) treatment of cell esds is used for estimating esds involving l.s. planes.

### Fractional atomic coordinates and isotropic or equivalent isotropic displacement parameters (Å^2^)

**Table:**

	*x*	*y*	*z*	*U*_iso_*/*U*_eq_	
Cd1	0.03947 (2)	0.26424 (2)	0.21210 (2)	0.00731 (4)	
Te1	0.000000	0.000000	0.500000	0.00444 (5)	
Te2	0.000000	0.000000	0.000000	0.00424 (5)	
O1	0.0289 (2)	0.1903 (2)	0.40560 (16)	0.0087 (2)	
O2	0.1800 (2)	0.1509 (2)	0.10570 (16)	0.0078 (2)	

### Atomic displacement parameters (Å^2^)

**Table:**

	*U*^11^	*U*^22^	*U*^33^	*U*^12^	*U*^13^	*U*^23^
Cd1	0.00657 (6)	0.00745 (6)	0.00789 (6)	0.00348 (5)	−0.00052 (4)	−0.00099 (4)
Te1	0.00419 (7)	0.00419 (7)	0.00492 (10)	0.00210 (3)	0.000	0.000
Te2	0.00403 (7)	0.00403 (7)	0.00464 (10)	0.00202 (3)	0.000	0.000
O1	0.0102 (6)	0.0074 (6)	0.0082 (5)	0.0043 (5)	−0.0001 (5)	0.0024 (4)
O2	0.0058 (5)	0.0069 (6)	0.0094 (6)	0.0021 (5)	−0.0024 (4)	−0.0017 (4)

### Geometric parameters (Å, º)

**Table:**

Cd1—O1	2.2348 (17)	Te1—O1^viii^	1.9339 (18)
Cd1—O2^i^	2.2455 (17)	Te1—O1^iii^	1.9339 (17)
Cd1—O1^ii^	2.2907 (19)	Te1—O1^ix^	1.9339 (17)
Cd1—O2^iii^	2.3051 (18)	Te1—O1^x^	1.9339 (17)
Cd1—O2	2.3370 (18)	Te1—O1^xi^	1.9339 (17)
Cd1—O1^iv^	2.4658 (19)	Te1—O1	1.9339 (17)
Cd1—Te2	3.2608 (2)	Te2—O2	1.9290 (17)
Cd1—Te1^v^	3.3420 (2)	Te2—O2^xii^	1.9290 (17)
Cd1—Cd1^ii^	3.3606 (3)	Te2—O2^iii^	1.9290 (17)
Cd1—Cd1^vi^	3.4239 (3)	Te2—O2^xiii^	1.9291 (17)
Cd1—Cd1^vii^	3.4537 (2)	Te2—O2^xiv^	1.9291 (17)
Cd1—Cd1^i^	3.4538 (2)	Te2—O2^xi^	1.9291 (17)
			
O1—Cd1—O2^i^	94.05 (7)	O1^x^—Te1—Cd1^xvii^	79.65 (6)
O1—Cd1—O1^ii^	84.10 (7)	O1^xi^—Te1—Cd1^xvii^	100.35 (6)
O2^i^—Cd1—O1^ii^	120.35 (7)	O1—Te1—Cd1^xvii^	138.37 (6)
O1—Cd1—O2^iii^	107.98 (7)	Cd1^xv^—Te1—Cd1^xvii^	117.776 (1)
O2^i^—Cd1—O2^iii^	82.41 (7)	Cd1^xvi^—Te1—Cd1^xvii^	62.224 (1)
O1^ii^—Cd1—O2^iii^	154.11 (6)	Cd1^ii^—Te1—Cd1^xvii^	180.0
O1—Cd1—O2	107.37 (6)	O1^viii^—Te1—Cd1^xviii^	100.35 (6)
O2^i^—Cd1—O2	148.88 (4)	O1^iii^—Te1—Cd1^xviii^	79.65 (6)
O1^ii^—Cd1—O2	84.88 (6)	O1^ix^—Te1—Cd1^xviii^	46.92 (5)
O2^iii^—Cd1—O2	69.79 (8)	O1^x^—Te1—Cd1^xviii^	138.37 (6)
O1—Cd1—O1^iv^	144.36 (6)	O1^xi^—Te1—Cd1^xviii^	41.63 (6)
O2^i^—Cd1—O1^iv^	82.90 (6)	O1—Te1—Cd1^xviii^	133.08 (5)
O1^ii^—Cd1—O1^iv^	67.58 (8)	Cd1^xv^—Te1—Cd1^xviii^	62.225 (1)
O2^iii^—Cd1—O1^iv^	106.78 (6)	Cd1^xvi^—Te1—Cd1^xviii^	117.775 (1)
O2—Cd1—O1^iv^	91.75 (6)	Cd1^ii^—Te1—Cd1^xviii^	117.775 (1)
O1—Cd1—Te2	119.57 (5)	Cd1^xvii^—Te1—Cd1^xviii^	62.225 (1)
O2^i^—Cd1—Te2	113.76 (5)	O1^viii^—Te1—Cd1^xix^	79.65 (6)
O1^ii^—Cd1—Te2	118.53 (5)	O1^iii^—Te1—Cd1^xix^	100.35 (6)
O2^iii^—Cd1—Te2	35.59 (4)	O1^ix^—Te1—Cd1^xix^	133.08 (5)
O2—Cd1—Te2	35.72 (4)	O1^x^—Te1—Cd1^xix^	41.63 (6)
O1^iv^—Cd1—Te2	93.60 (4)	O1^xi^—Te1—Cd1^xix^	138.37 (6)
O1—Cd1—Te1^v^	111.58 (5)	O1—Te1—Cd1^xix^	46.92 (5)
O2^i^—Cd1—Te1^v^	97.31 (5)	Cd1^xv^—Te1—Cd1^xix^	117.775 (1)
O1^ii^—Cd1—Te1^v^	34.11 (4)	Cd1^xvi^—Te1—Cd1^xix^	62.225 (1)
O2^iii^—Cd1—Te1^v^	140.34 (4)	Cd1^ii^—Te1—Cd1^xix^	62.225 (1)
O2—Cd1—Te1^v^	95.47 (4)	Cd1^xvii^—Te1—Cd1^xix^	117.775 (1)
O1^iv^—Cd1—Te1^v^	34.95 (4)	Cd1^xviii^—Te1—Cd1^xix^	180.0
Te2—Cd1—Te1^v^	116.088 (5)	O2—Te2—O2^xii^	93.00 (8)
O1—Cd1—Cd1^ii^	42.69 (5)	O2—Te2—O2^iii^	87.00 (8)
O2^i^—Cd1—Cd1^ii^	113.04 (5)	O2^xii^—Te2—O2^iii^	180.00 (11)
O1^ii^—Cd1—Cd1^ii^	41.41 (4)	O2—Te2—O2^xiii^	180.0
O2^iii^—Cd1—Cd1^ii^	144.94 (5)	O2^xii^—Te2—O2^xiii^	87.00 (8)
O2—Cd1—Cd1^ii^	97.92 (4)	O2^iii^—Te2—O2^xiii^	93.00 (8)
O1^iv^—Cd1—Cd1^ii^	106.29 (4)	O2—Te2—O2^xiv^	93.00 (8)
Te2—Cd1—Cd1^ii^	130.823 (8)	O2^xii^—Te2—O2^xiv^	87.00 (8)
Te1^v^—Cd1—Cd1^ii^	71.352 (5)	O2^iii^—Te2—O2^xiv^	93.00 (8)
O1—Cd1—Cd1^vi^	104.72 (5)	O2^xiii^—Te2—O2^xiv^	87.00 (8)
O2^i^—Cd1—Cd1^vi^	41.86 (5)	O2—Te2—O2^xi^	87.00 (8)
O1^ii^—Cd1—Cd1^vi^	159.57 (5)	O2^xii^—Te2—O2^xi^	93.00 (8)
O2^iii^—Cd1—Cd1^vi^	40.55 (4)	O2^iii^—Te2—O2^xi^	87.00 (8)
O2—Cd1—Cd1^vi^	109.20 (4)	O2^xiii^—Te2—O2^xi^	93.00 (8)
O1^iv^—Cd1—Cd1^vi^	96.51 (4)	O2^xiv^—Te2—O2^xi^	180.00 (13)
Te2—Cd1—Cd1^vi^	73.547 (5)	O2—Te2—Cd1^xiii^	134.98 (5)
Te1^v^—Cd1—Cd1^vi^	126.995 (8)	O2^xii^—Te2—Cd1^xiii^	44.06 (5)
Cd1^ii^—Cd1—Cd1^vi^	143.877 (10)	O2^iii^—Te2—Cd1^xiii^	135.94 (5)
O1—Cd1—Cd1^vii^	104.17 (5)	O2^xiii^—Te2—Cd1^xiii^	45.02 (5)
O2^i^—Cd1—Cd1^vii^	153.98 (5)	O2^xiv^—Te2—Cd1^xiii^	96.56 (5)
O1^ii^—Cd1—Cd1^vii^	45.47 (5)	O2^xi^—Te2—Cd1^xiii^	83.44 (5)
O2^iii^—Cd1—Cd1^vii^	108.67 (4)	O2—Te2—Cd1^xi^	44.06 (5)
O2—Cd1—Cd1^vii^	40.10 (4)	O2^xii^—Te2—Cd1^xi^	83.44 (5)
O1^iv^—Cd1—Cd1^vii^	71.44 (4)	O2^iii^—Te2—Cd1^xi^	96.56 (5)
Te2—Cd1—Cd1^vii^	73.148 (6)	O2^xiii^—Te2—Cd1^xi^	135.94 (5)
Te1^v^—Cd1—Cd1^vii^	58.887 (1)	O2^xiv^—Te2—Cd1^xi^	134.98 (5)
Cd1^ii^—Cd1—Cd1^vii^	71.628 (5)	O2^xi^—Te2—Cd1^xi^	45.02 (5)
Cd1^vi^—Cd1—Cd1^vii^	143.600 (10)	Cd1^xiii^—Te2—Cd1^xi^	106.152 (5)
O1—Cd1—Cd1^i^	122.01 (5)	O2—Te2—Cd1	45.02 (5)
O2^i^—Cd1—Cd1^i^	42.10 (5)	O2^xii^—Te2—Cd1	135.94 (5)
O1^ii^—Cd1—Cd1^i^	90.20 (4)	O2^iii^—Te2—Cd1	44.06 (5)
O2^iii^—Cd1—Cd1^i^	101.62 (5)	O2^xiii^—Te2—Cd1	134.98 (5)
O2—Cd1—Cd1^i^	129.56 (4)	O2^xiv^—Te2—Cd1	83.44 (5)
O1^iv^—Cd1—Cd1^i^	41.48 (4)	O2^xi^—Te2—Cd1	96.56 (5)
Te2—Cd1—Cd1^i^	113.622 (7)	Cd1^xiii^—Te2—Cd1	180.0
Te1^v^—Cd1—Cd1^i^	58.888 (1)	Cd1^xi^—Te2—Cd1	73.847 (5)
Cd1^ii^—Cd1—Cd1^i^	110.788 (6)	O2—Te2—Cd1^xiv^	135.94 (5)
Cd1^vi^—Cd1—Cd1^i^	69.450 (8)	O2^xii^—Te2—Cd1^xiv^	96.56 (5)
Cd1^vii^—Cd1—Cd1^i^	111.882 (5)	O2^iii^—Te2—Cd1^xiv^	83.44 (5)
O1^viii^—Te1—O1^iii^	180.0	O2^xiii^—Te2—Cd1^xiv^	44.06 (5)
O1^viii^—Te1—O1^ix^	93.54 (7)	O2^xiv^—Te2—Cd1^xiv^	45.02 (5)
O1^iii^—Te1—O1^ix^	86.46 (7)	O2^xi^—Te2—Cd1^xiv^	134.98 (5)
O1^viii^—Te1—O1^x^	93.54 (7)	Cd1^xiii^—Te2—Cd1^xiv^	73.848 (5)
O1^iii^—Te1—O1^x^	86.46 (7)	Cd1^xi^—Te2—Cd1^xiv^	180.0
O1^ix^—Te1—O1^x^	93.54 (7)	Cd1—Te2—Cd1^xiv^	106.153 (5)
O1^viii^—Te1—O1^xi^	86.46 (7)	O2—Te2—Cd1^xii^	83.44 (5)
O1^iii^—Te1—O1^xi^	93.54 (7)	O2^xii^—Te2—Cd1^xii^	45.02 (5)
O1^ix^—Te1—O1^xi^	86.46 (7)	O2^iii^—Te2—Cd1^xii^	134.98 (5)
O1^x^—Te1—O1^xi^	180.0	O2^xiii^—Te2—Cd1^xii^	96.56 (5)
O1^viii^—Te1—O1	86.46 (7)	O2^xiv^—Te2—Cd1^xii^	44.06 (5)
O1^iii^—Te1—O1	93.54 (7)	O2^xi^—Te2—Cd1^xii^	135.94 (5)
O1^ix^—Te1—O1	180.0	Cd1^xiii^—Te2—Cd1^xii^	73.848 (5)
O1^x^—Te1—O1	86.46 (7)	Cd1^xi^—Te2—Cd1^xii^	106.152 (5)
O1^xi^—Te1—O1	93.54 (7)	Cd1—Te2—Cd1^xii^	106.153 (5)
O1^viii^—Te1—Cd1^xv^	41.63 (6)	Cd1^xiv^—Te2—Cd1^xii^	73.848 (5)
O1^iii^—Te1—Cd1^xv^	138.37 (6)	O2—Te2—Cd1^iii^	96.56 (5)
O1^ix^—Te1—Cd1^xv^	79.65 (6)	O2^xii^—Te2—Cd1^iii^	134.98 (5)
O1^x^—Te1—Cd1^xv^	133.08 (6)	O2^iii^—Te2—Cd1^iii^	45.02 (5)
O1^xi^—Te1—Cd1^xv^	46.92 (6)	O2^xiii^—Te2—Cd1^iii^	83.44 (5)
O1—Te1—Cd1^xv^	100.35 (6)	O2^xiv^—Te2—Cd1^iii^	135.94 (5)
O1^viii^—Te1—Cd1^xvi^	138.37 (6)	O2^xi^—Te2—Cd1^iii^	44.06 (5)
O1^iii^—Te1—Cd1^xvi^	41.63 (6)	Cd1^xiii^—Te2—Cd1^iii^	106.152 (5)
O1^ix^—Te1—Cd1^xvi^	100.35 (6)	Cd1^xi^—Te2—Cd1^iii^	73.848 (5)
O1^x^—Te1—Cd1^xvi^	46.92 (6)	Cd1—Te2—Cd1^iii^	73.847 (5)
O1^xi^—Te1—Cd1^xvi^	133.08 (6)	Cd1^xiv^—Te2—Cd1^iii^	106.152 (5)
O1—Te1—Cd1^xvi^	79.65 (6)	Cd1^xii^—Te2—Cd1^iii^	180.000 (11)
Cd1^xv^—Te1—Cd1^xvi^	180.0	Te1—O1—Cd1	139.23 (10)
O1^viii^—Te1—Cd1^ii^	46.92 (5)	Te1—O1—Cd1^ii^	104.25 (8)
O1^iii^—Te1—Cd1^ii^	133.08 (5)	Cd1—O1—Cd1^ii^	95.90 (7)
O1^ix^—Te1—Cd1^ii^	138.37 (6)	Te1—O1—Cd1^xix^	98.13 (7)
O1^x^—Te1—Cd1^ii^	100.35 (6)	Cd1—O1—Cd1^xix^	116.00 (7)
O1^xi^—Te1—Cd1^ii^	79.65 (6)	Cd1^ii^—O1—Cd1^xix^	93.05 (7)
O1—Te1—Cd1^ii^	41.63 (6)	Te2—O2—Cd1^vii^	147.03 (10)
Cd1^xv^—Te1—Cd1^ii^	62.223 (1)	Te2—O2—Cd1^xi^	100.35 (7)
Cd1^xvi^—Te1—Cd1^ii^	117.776 (1)	Cd1^vii^—O2—Cd1^xi^	97.59 (7)
O1^viii^—Te1—Cd1^xvii^	133.08 (5)	Te2—O2—Cd1	99.25 (7)
O1^iii^—Te1—Cd1^xvii^	46.92 (5)	Cd1^vii^—O2—Cd1	97.80 (7)
O1^ix^—Te1—Cd1^xvii^	41.63 (6)	Cd1^xi^—O2—Cd1	115.12 (8)

Symmetry codes: (i) *y*−1/3, −*x*+*y*+1/3, −*z*+1/3; (ii) −*x*+1/3, −*y*+2/3, −*z*+2/3; (iii) −*y*, *x*−*y*, *z*; (iv) −*y*+1/3, *x*−*y*+2/3, *z*−1/3; (v) *x*+1/3, *y*+2/3, *z*−1/3; (vi) −*x*−1/3, −*y*+1/3, −*z*+1/3; (vii) *x*−*y*+2/3, *x*+1/3, −*z*+1/3; (viii) *y*, −*x*+*y*, −*z*+1; (ix) −*x*, −*y*, −*z*+1; (x) *x*−*y*, *x*, −*z*+1; (xi) −*x*+*y*, −*x*, *z*; (xii) *y*, −*x*+*y*, −*z*; (xiii) −*x*, −*y*, −*z*; (xiv) *x*−*y*, *x*, −*z*; (xv) −*y*+2/3, *x*−*y*+1/3, *z*+1/3; (xvi) *y*−2/3, −*x*+*y*−1/3, −*z*+2/3; (xvii) *x*−1/3, *y*−2/3, *z*+1/3; (xviii) *x*−*y*+1/3, *x*−1/3, −*z*+2/3; (xix) −*x*+*y*−1/3, −*x*+1/3, *z*+1/3.
